# Respiratory variations in pulse pressure and photoplethysmographic waveform amplitude during positive expiratory pressure and continuous positive airway pressure in a model of progressive hypovolemia

**DOI:** 10.1371/journal.pone.0223071

**Published:** 2019-09-27

**Authors:** Ingrid Elise Hoff, Jonny Hisdal, Svein Aslak Landsverk, Jo Røislien, Knut Arvid Kirkebøen, Lars Øivind Høiseth

**Affiliations:** 1 Norwegian Air Ambulance Foundation, Sentrum, Oslo, Norway; 2 Department of Anesthesiology, Oslo University Hospital, Nydalen, Oslo, Norway; 3 Section of Vascular Investigations, Department of Vascular Surgery, Oslo University Hospital, Nydalen, Oslo, Norway; 4 Faculty of Medicine, University of Oslo, Blindern, Oslo, Norway; Scuola Superiore Sant’Anna, ITALY

## Abstract

**Purpose:**

Respiratory variations in pulse pressure (dPP) and photoplethysmographic waveform amplitude (dPOP) are used for evaluation of volume status in mechanically ventilated patients. Amplification of intrathoracic pressure changes may enable their use also during spontaneous breathing. We investigated the association between the degree of hypovolemia and dPP and dPOP at different levels of two commonly applied clinical interventions; positive expiratory pressure (PEP) and continuous positive airway pressure (CPAP).

**Methods:**

20 healthy volunteers were exposed to progressive hypovolemia by lower body negative pressure (LBNP). PEP of 0 (baseline), 5 and 10 cmH_2_O was applied by an expiratory resistor and CPAP of 0 (baseline), 5 and 10 cmH_2_O by a facemask. dPP was obtained non-invasively with the volume clamp method and dPOP from a pulse oximeter. Central venous pressure was measured in 10 subjects. Associations between changes were examined using linear mixed-effects regression models.

**Results:**

dPP increased with progressive LBNP at all levels of PEP and CPAP. The LBNP-induced increase in dPP was amplified by PEP 10 cmH_2_0. dPOP increased with progressive LBNP during PEP 5 and PEP 10, and during all levels of CPAP. There was no additional effect of the level of PEP or CPAP on dPOP. Progressive hypovolemia and increasing levels of PEP were reflected by increasing respiratory variations in CVP.

**Conclusion:**

dPP and dPOP reflected progressive hypovolemia in spontaneously breathing healthy volunteers during PEP and CPAP. An increase in PEP from baseline to 10 cmH_2_O augmented the increase in dPP, but not in dPOP.

## Introduction

Dynamic variables, such as respiratory variations in pulse pressure (dPP) and the photoplethysmographic waveform amplitude (dPOP), are accurate indicators of preload changes, and predict fluid responsiveness during mechanical ventilation [1–3]. During mechanical ventilation, dPP is mainly caused by increased intrathoracic pressure in the inspiratory phase, leading to increased right atrial pressure (RAP). As RAP, which normally equals central venous pressure (CVP) [4], is the pressure opposing venous return, this leads to cyclic variations in venous return and thus stroke volume and pulse pressure, which are larger when the heart is preload responsive. It is generally assumed that these variations are inadequate to enable dynamic variables to reflect preload dependency in spontaneously breathing subjects. However, some studies indicate that the ability of dPP and dPOP to reflect volume status or predict fluid responsiveness improves when intrathoracic pressure variations are amplified with respiratory resistance [5, 6].

Positive expiratory pressure (PEP) and continuous positive airway pressure (CPAP) are two clinical interventions which increase airway pressure and are frequently used to prevent atelectasis and respiratory failure in spontaneously breathing patients postoperatively and during critical illness. In these patients, evaluation of volume status is important, and whether PEP and CPAP may affect the ability of dPP and dPOP to detect hypovolemia is of clinical relevance, as the use of these respiratory interventions could also provide an opportunity to evaluate volume status.

This study aimed to investigate the ability of dPP and dPOP to track hypovolemia induced by lower body negative pressure (LBNP) in spontaneously breathing volunteers during different levels of PEP and CPAP. We further investigated whether associations between the dynamic variables and volume status were affected by the level of respiratory resistance. We also aimed to explore whether level of respiratory resistance was reflected in the respiratory variations in CVP (dCVP). We hypothesized that the dynamic variables would increase with progressive hypovolemia and that the increases would be amplified with increasing levels of respiratory resistance.

## Methods

### Subjects

The study was approved by the Regional Committee for Medical and Health Research Ethics (REC South East D, reference 2015/344) prior to inclusion. All procedures were in accordance with the ethical standards of the institutional and regional ethical research committees and with the 1964 Helsinki declaration and its later amendments or comparable ethical standards. Twenty healthy adult volunteers (11 males, 9 females, aged 25(3) (mean[SD]) years, height 176(9) cm and weight 69(8) kg were included from November 2015 to December 2016 after oral and written informed consent. Exclusion criteria were disease or disability requiring regular medication (except allergies), arrhythmia, pregnancy, history of syncope, and infection in the elbow crease. Participants refrained from caffeine consumption and excessive physical exercise on the day of the experiments.

### Study protocol

**Fig 1:**
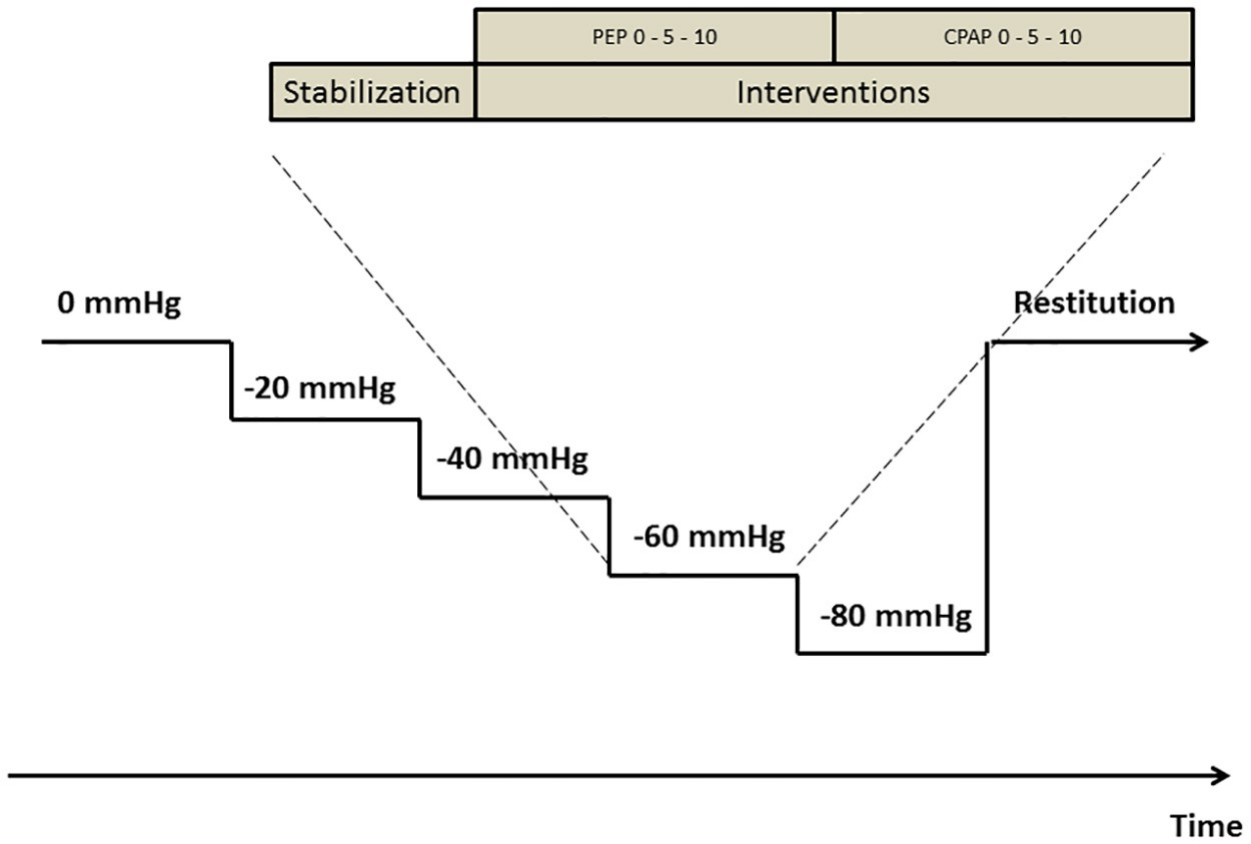
Protocol. Each LBNP-level lasted for approximately 6 min and consisted of 1 min stabilization and minimum 5 breaths at each level of PEP and CPAP. PEP- and CPAP-levels were assigned in randomized order. *PEP*: positive expiratory pressure, *CPAP*: continuous positive airway pressure.

The experiments were performed between 8:00 AM and 4:00 PM in a vascular investigation lab air-conditioned to 20–22°C. Fig 1 illustrates the study protocol. Subjects were placed in the supine position in the LBNP chamber, which was sealed with a neoprene skirt at the level of the iliac crest as described previously [7]. LBNP leads to sequestering of blood in the lower abdomen and extremities, with a negative pressure of 80 mmHg corresponding to a blood loss of more than 1 liter [8]. Stepwise progressions in negative pressure from 0 (baseline) to 20, 40, 60 and 80 mmHg were induced. Each LBNP-level lasted approximately 6 min. At each LBNP-level, after 1 min allowing the stabilization of hemodynamic values, PEP of 0 (baseline), 5 and 10 cmH_2_O was applied, followed by CPAP of 0 (baseline), 5 and 10 cmH_2_O in alternating order at different LBNP-levels. Subjects were randomized in blocks of six generated by pseudorandom numbers in Excel 2010 (Microsoft Office 365; Microsoft Corporation, Redmond, Washington, USA) to start with different PEP or CPAP levels, thus also changing the order at the subsequent LBNP-levels. PEP was induced with an expiratory resistor (Armstrong Medical Ltd, Coleraine, Northern Ireland), through which the subjects were instructed to exhale calmly. CPAP was provided by Drӓger Evita 4 (Drӓgerwerk, Lübeck, Germany), and the subjects were instructed to breathe normally in the facemask. No instructions were given for respiratory rate, allowing the subjects to adjust the ventilation to avoid hyper–and hypoventilation. PEP 0 cmH_2_0 was breathing through an empty mouthpiece, and CPAP 0 cmH_2_O was breathing through a facemask with CPAP set to 0 cmH_2_O on the ventilator. Each pressure level was applied for 5–6 breaths. LBNP was released and the protocol terminated at the subject`s request, or if the subject displayed signs of impending circulatory collapse such as sweating, nausea or dizziness or a sudden marked reduction in heart rate or mean arterial pressure. Only measurements from completed breathing sequences were used for analyses.

### Data acquisition

Non-invasive arterial pressure waveform was acquired by the volume-clamp method (Finometer; FMS Finapres Mesurement Systems, Arnhem, The Netherlands) and exported with ECG at 300 Hz to custom-made software (Regist3; Morten Eriksen, University of Oslo, Oslo, Norway). Photoplethysmographic waveform from a commercially available pulse oximeter attached to the right 2. finger (Masimo Radical 7, software 7.3.1.1, Masimo Corp., Irvine, CA, USA with probe LNOP DC-I; Masimo Corp.) was exported from the analog output at 400 Hz to SignalExpress 14.0.0 (National Instruments, Austin, Texas, USA). Aortic blood flow velocity was measured by suprasternal Doppler with a 2 MHz probe (SD-50; GE Vingmed Ultrasound, Horten, Norway) and sampled at 300 Hz in Regist3. In 10 subjects, CVP was obtained from a central venous catheter (Secalon Seldy 16G, Argon Critical Care Systems, Singapore), which was inserted via the left basilic vein to the left subclavian vein and connected to a pressure transducer (CODAN Critical Care GmbH, Forstinning, Germany), which was leveled and zeroed in the mid-axillary line. Correct position was verified by a typical central venous pressure waveform. CVP-measurements from peripherally inserted catheters and conventional central venous catheters have been shown to be highly correlated [9]. CVP waveforms were exported with ECG from the TramRac4A (General Electric Healthcare) at 400 Hz to SignalExpress. Analog signals were exported as text files, time synchronized and handled in R 3.4.0 (R Foundation for Statistical Computing, Vienna, Austria) using RStudio 1.0.143 (RStudio, Boston, MA, USA).

### Signal analysis and calculations

Stroke volume was obtained by calculating aortic flow velocity-time integrals gated by the R-peaks of the ECG, assuming an angle of 20° to the aortic blood flow and an aortic diameter of 20 mm [10]. Cardiac output was calculated by multiplying stroke volume with heart rate. dPP and dPOP were calculated by the formulas

$$\Delta PP=\frac{PP\;max-PPmin}{\frac{PPmax+PPmin}{2}}\times 100\%$$

and

$$\Delta POP=\frac{POP\;max-POPmin}{\frac{POPmax+POPmin}{2}}\times 100\%,$$

where PP_max_ and PP_min_ are the maximal and minimal pulse pressures and POP_max_ and POP_min_ are the maximal and minimal photoplethysmographic amplitudes within one respiratory cycle. Calculations were performed in R using the WaveletComp [11] and peakPick-packages [12]. After downsampling to 40 Hz, respiratory variations in CVP (dCVP) were calculated as the absolute difference between the peak and trough of the CVP-pressure waveform within one respiratory cycle. All calculated values were plotted and visually inspected before being accepted to the final dataset. Respiratory cycles with obvious disturbances (e.g. motion artifacts) were omitted. Details and examples of the calculations are presented in S1 and S2 Files. For stroke volume, heart rate, mean arterial pressure and CVP, mean values, trimming the highest and lowest 5% to remove disturbances, were calculated for each LBNP-level.

### Statistical analyses

Sample size was estimated for another protocol performed on the same subjects the same day, and a separate power analysis was not performed for the currently presented results. The number of subjects in the present study is comparable to other studies published in the same field. Confidence intervals of the present analyses are displayed according to the CONSORT guidelines [13].

The potential effects of PEP and CPAP on the dynamic variables were analyzed separately. The associations between the level of respiratory resistance and LBNP (explanatory variables), and the dynamic variables (outcome variables) were explored using linear mixed models (LMM) due to the clustering of data within subjects. Level of respiratory resistance (baseline, 5 or 10 cmH_2_0) was treated as a factor and LBNP-level as a continuous variable, including an interaction term between the two. When plotting LBNP-level on the x-axis and a dynamic variable on the y-axis, the slope represents the change in dynamic variable with a change in volume status, and thus the ability to reflect changes in volume status. The dynamic variables were right-skewed on the original scale, and were therefore log_e_-transformed before analysis. The results are presented back-transformed in the figures for clarity. Data were analyzed in R using RStudio. LMM was fitted using the glmmPQL-function of “MASS” package [14], and estimates with confidence intervals for each LBNP and respiratory resistance level were calculated using the “glht”- function of the “multcomp”-package [15]. P-values were corrected for multiple post-hoc comparisons by the “single-step” method in the “multcomp” package. P-values < 0.05 were considered statistically significant.

## Results

All subjects completed LBNP 20 mmHg, 19 subjects completed LBNP 40 mmHg, 13 subjects completed LBNP 60 mmHg and five subjects completed LBNP 80 mmHg. Each LBNP-level lasted 6.0 (5.3, 6.7) min (median [25th, 75th percentiles], and the entire LBNP-exposure (from LBNP 20 mmHg) lasted 18 (14, 22) min. Overall the respiratory rate during PEP was 14.0 (11.4, 16.6) and during CPAP 13.1 (9.9, 15.0) breaths /min.

**Fig 2:**
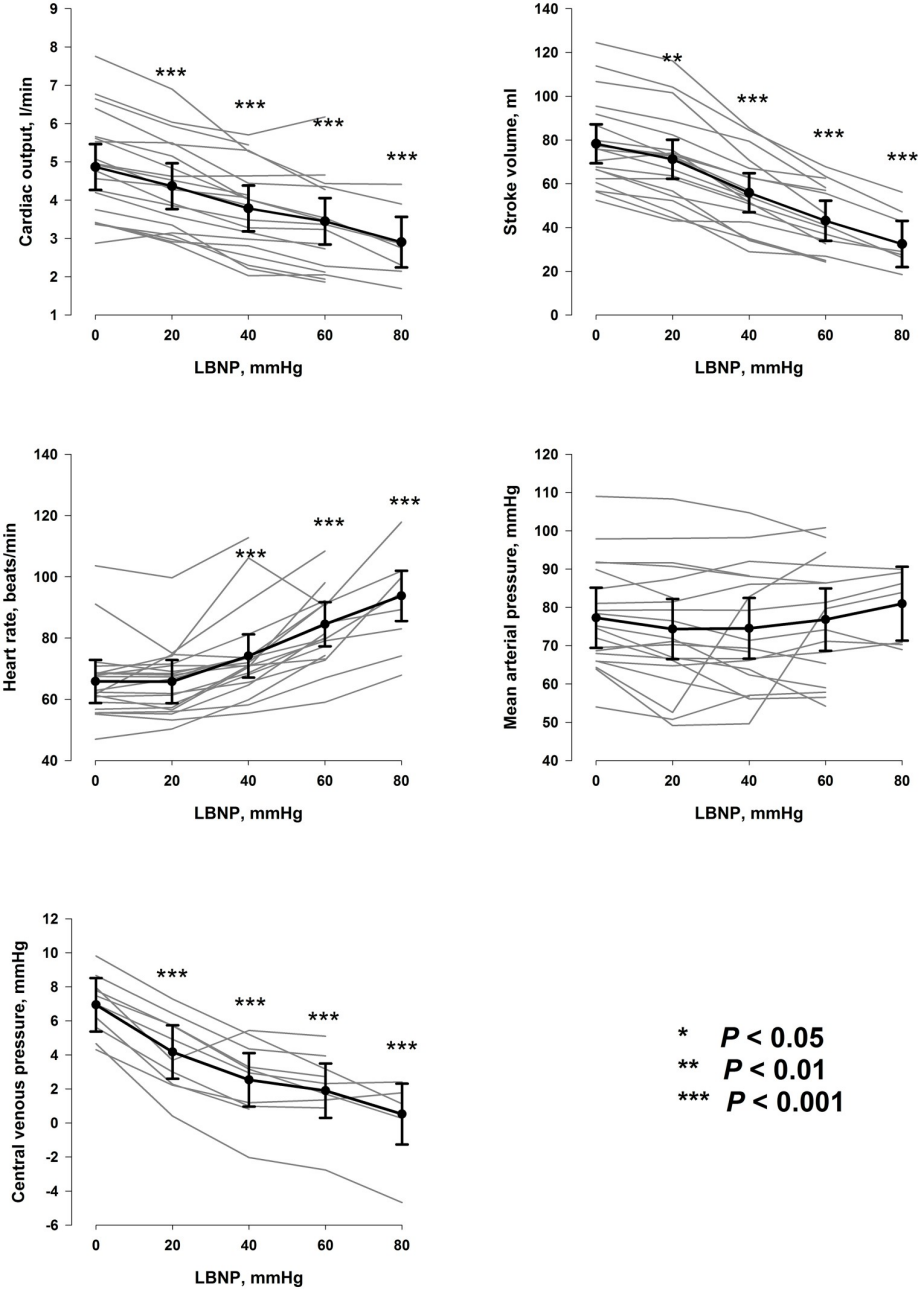
Hemodynamic data. Individual hemodynamic data (grey lines) and estimates from the regression models (black lines, with 95% confidence intervals) for each level of LBNP. P-values are compared to LBNP 0 mmHg. *LBNP*: lower body negative pressure.

Hemodynamic data are shown in Fig 2. Stroke volume, cardiac output and CVP were reduced from LBNP 0 mmHg at all LBNP-levels, whereas heart rate was increased from LBNP 40 mmHg. Mean arterial pressure did not change with progressive LBNP.

**Fig 3:**
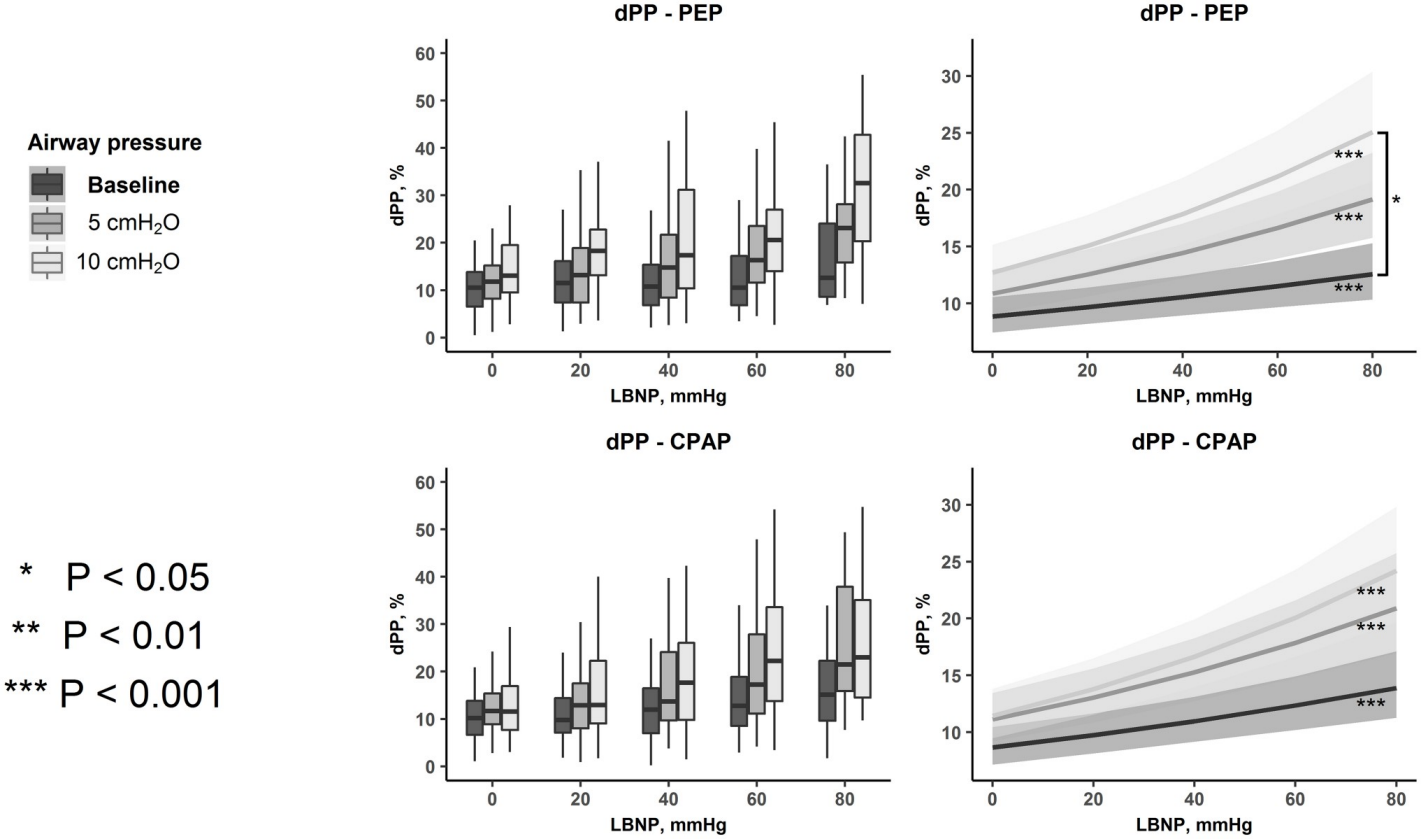
Respiratory variations in pulse pressure (dPP) during PEP and CPAP. The effects of LBNP and level of respiratory resistance on dPP. Boxplots of respiratory variations in PP from all observations in all subjects at each level of LBNP and respiratory resistance (left panel). Lines with ribbons are estimates and confidence intervals from the regression models (right panel). The slopes of the regression lines represent the change of dPP with a change in LBNP. Note that as the linear regressions were performed on the log_e_-values of dPP, the lines are curved on the original scale, as presented in the figure. *dPP*: respiratory variations in pulse pressure, *PEP*: positive expiratory pressure, *CPAP*: continuous positive airway pressure, *LBNP*: lower body negative pressure.

**Fig 4:**
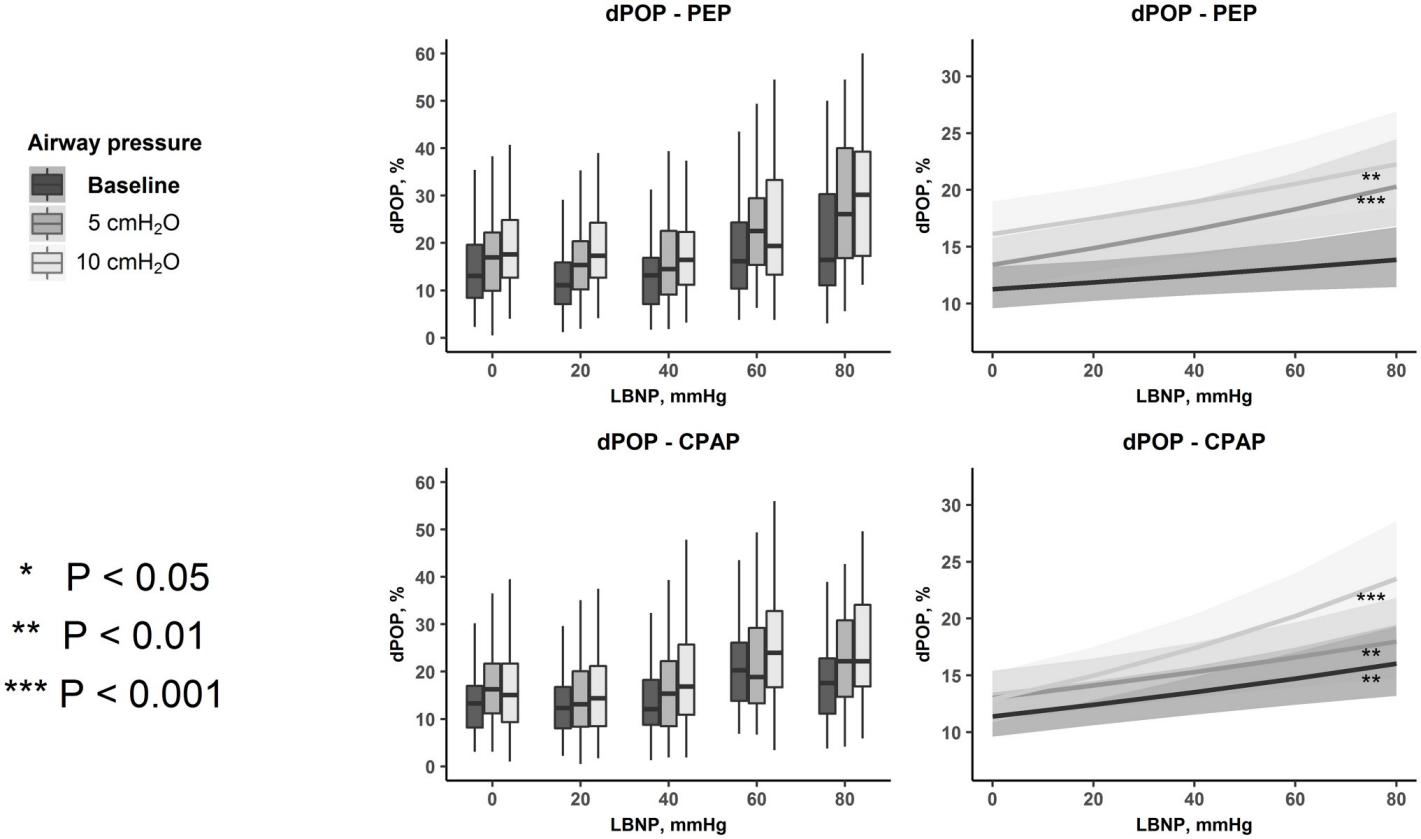
Respiratory variations in the photoplethysmographic waveform amplitude (dPOP) during PEP and CPAP. The effects of LBNP and level of respiratory resistance on dPOP. Boxplots of respiratory variations in POP from all observations in all subjects at each level of LBNP and respiratory resistance (left panel). Lines with ribbons are estimates and confidence intervals from the regression models (right panel). The slopes of the regression lines represent the change of dPOP with a change in LBNP. Note that as the linear regressions were performed on the log_e_-values of dPOP, the lines are curved on the original scale, as presented in the figure. *dPOP*: respiratory variations in the photoplethysmographic waveform, *PEP*: positive expiratory pressure, *CPAP*: continuous positive airway pressure, *LBNP*: lower body negative pressure.

dPP and dPOP with progressive LBNP and different levels of PEP or CPAP are presented in Figs 3 and 4. dPP increased significantly with progressive LBNP both at baseline and during higher levels of PEP and CPAP. dPOP did not significantly increase with progressive LBNP during baseline PEP, but during PEP 5 and 10 cmH_2_O, and during all levels of CPAP. There was a significant difference between the regression slopes of dPP at baseline PEP and PEP 10 cmH_2_0, indicating that the increase in dPP with progressive LBNP was amplified by the application of PEP 10 cmH_2_0. The application of PEP 5 cmH_2_0, CPAP 5 or 10 cmH_2_0 did not induce any additional changes in dPP or dPOP (Table 1).

**Table 1:** Effects of LBNP-, PEP- and CPAP-level on dPP and dPOP.

Intervention	PEP-/CPAP-level in cm H_2_O		Slope coefficient (95% CI)	*P*-value	Difference to 0 cmH_2_0 (95% CI)	*P*-value	Difference to 5cmH_2_0 (95% CI)	*P*-value
**PEP**	**0**	**Log**_**e**_ **(dPP)**	0.088 (0.032 to 0.14)	<0.001				
	**5**		0.14 (0.087 to 0.20)	<0.001	0.054 (-0.023 to 0.13)	0.27		
	**10**		0.17 (0.12 to 0.22)	<0.001	0.082 (0.006 to 0.16)	0.028	0.028 (-0.047 to 0.10)	0.76
	**0**	**Log**_**e**_**(dPOP)**	0.051 (-0.01 to 0.11)	0.13				
	**5**		0.10 (0.043 to 0.17)	<0.001	0.052 (-0.033 to 0.14)	0.38		
	**10**		0.08 (0.019 to 0.14)	0.004	0.029 (-0.057 to 0.12)	0.81	-0.023(-0.11 to 0.062)	0.89
**CPAP**	**0**	**Log**_**e**_ **(dPP)**	0.12 (0.062 to 0.17)	<0.001				
	**5**		0.16 (0.10 to 0.21)	<0.001	0.039 (-0.038 to 0.12)	0.55		
	**10**		0.19 (0.13 to 0.24)	<0.001	0.069 (-0.008 to 0.15)	0.097	0.03 (-0.047 to 0.11)	0.74
	**0**	**Log**_**e**_**(dPOP)**	0.086 (0.025 to 0.15)	<0.002				
	**5**		0.08 (0.02 to 0.14)	<0.004	-0.005 (-0.088 to 0.078)	1.0		
	**10**		0.15 (0.09 to 0.21)	<0.001	0.065 (0.019 to 0.15)	0.19	0.069 (-0.014 to 0.15)	0.14

The slope coefficients of dPP and dPOP for each LBNP-level (0, 20, 40, 60 and 80) during different levels of PEP and CPAP, and comparisons between the PEP and CPAP levels. dPP and dPOP-data are log_e_-transformed due to right skewness of the residuals. *dPP*: respiratory variations in pulse pressure, *dPOP*: respiratory variations in the photoplethysmographic waveform amplitude. *LBNP*: lower body negative pressure, *PEP*: positive expiratory pressure, *CPAP*: continuous positive airway pressure.

**Fig 5:**
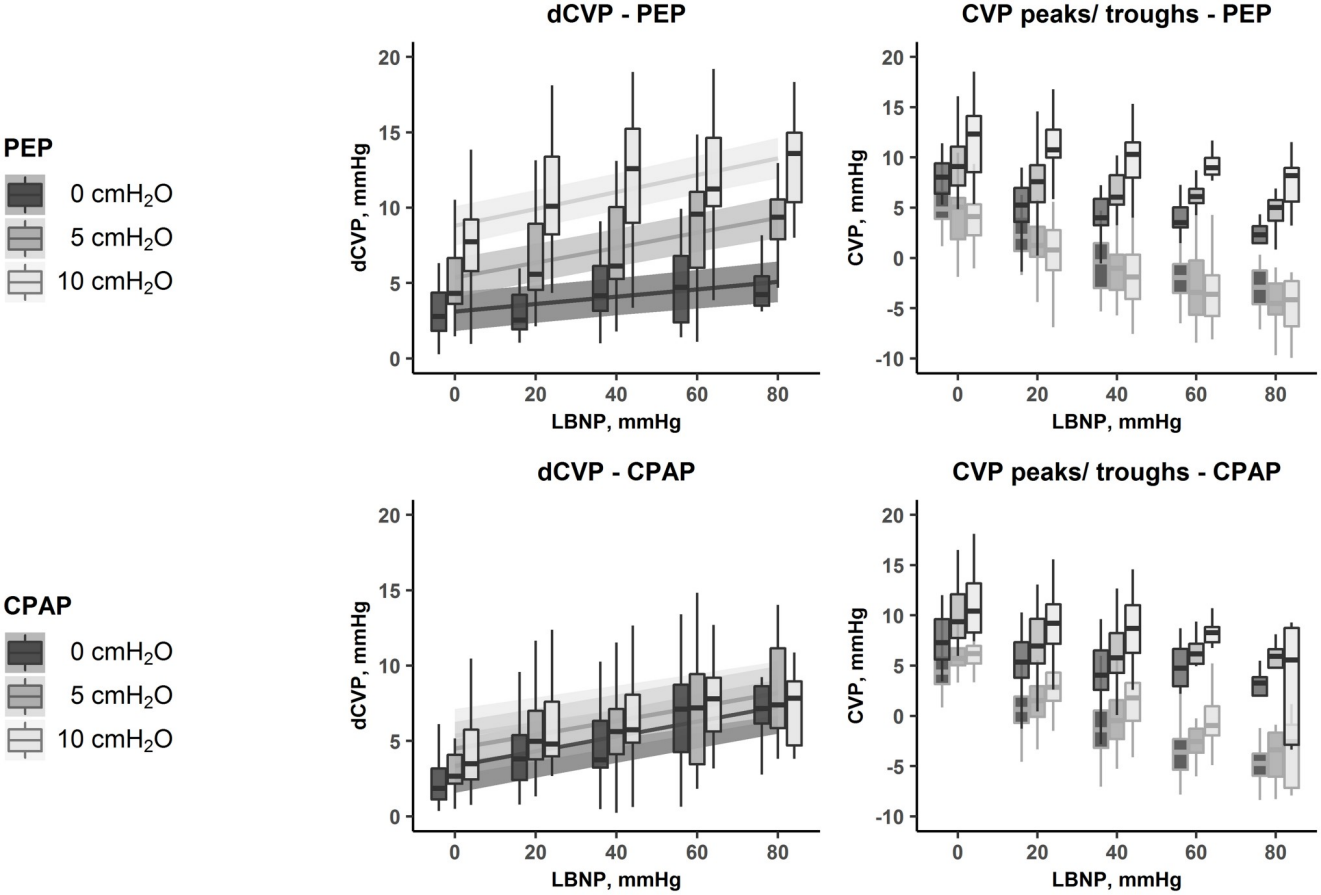
Respiratory variations in central venous pressure (dCVP) during PEP and CPAP. The effects of LBNP and level of respiratory resistance on CVP. Boxplots are from all observations in all subjects. Left panels show respiratory variations (difference between maximal and minimal CVP within each respiratory cycle) with different LBNP and PEP-/CPAP-levels. Right panels show the peaks and troughs within each respiratory cycle. The difference between the peaks and troughs gives the respiratory variations seen in the left panels. The lines with ribbons behind the boxplots are estimates and confidence intervals from the regression models. *CVP*: central venous pressure, *dCVP*: respiratory variations in central venous pressure, *PEP*: positive expiratory pressure, CPAP: continuous positive airway pressure, *LBNP*: lower body negative pressure.

dCVP increased significantly with increasing LBNP during all levels of PEP and CPAP (Table 2 and Fig 5). The increase in dCVP for any LBNP-level was significantly larger during PEP 5 cmH_2_0 and PEP 10 cmH_2_0 than during baseline PEP. By contrast, change in CPAP-level did not amplify the increase in dCVP.

**Table 2:** Effects of LBNP-, PEP- and CPAP-level on dCVP.

Intervention	PEP-/CPAP-level in cm H_2_O		Slope coefficient (95% CI)	*P*-value	Difference to 0 cmH_2_0 (95% CI)	*P*-value	Difference to 5cmH_2_0 (95% CI)	*P*-value
**PEP**	**0**	**dCVP**	0.49 (0.18 to 0.80)	<0.001				
	**5**		1.0 (0.68 to 1.3)	<0.001	0.50 (0.063 to 0.94)	0.018		
	**10**		1.1 (0.81 to 1.43)	<0.001	0.63 (0.20 to 1.1)	0.001	0.13 (-0.31 to 0.56)	0.88
**CPAP**	**0**	**dCVP**	0.99 (0.75 to 1.2)	<0.001				
	**5**		0.92 (0.69 to 1.2)	<0.001	-0.063 (0.39 to 0.26)	0.96		
	**10**		0.78 (0.55 to 1.0)	<0.001	-0.21 (-0.53 to 0.12)	0.35	-0.14 (-0.47 to 0.18)	0.66

The slope coefficient of dCVP for each LBNP-level (0, 20, 40, 60 and 80) during different levels of PEP and CPAP, and comparisons between the PEP and CPAP levels. *dCVP*: respiratory variations in central venous pressure, *LBNP*: lower body negative pressure, *PEP*: positive expiratory pressure, *CPAP*: continuous positive airway pressure.

## Discussion

The main findings of this experimental study were that the dynamic variables dPP and dPOP reflected progressive hypovolemia during all levels of CPAP and PEP, except for dPOP at baseline PEP. The application of PEP 10 cmH_2_O amplified the increase in dPP, but not in dPOP. Both progressive LBNP and the transition from baseline PEP to PEP 5 cmH_2_O and 10 cmH_2_O were associated with increases in dCVP.

During mechanical ventilation, several mechanisms contribute to respiratory induced changes in stroke volume [16]. The most important for the use of dynamic variables to diagnose volume status is the reduction in the gradient for venous return as RAP increases in the inspiratory phase. During mechanical ventilation, studies have shown that higher tidal volumes lead to an increase in dPP and SVV [17, 18]. Mesquida et al. altered tidal volume, chest wall compliance and cardiac function in dogs, and found that all changes in SVV and dPP were due to changes in right ventricular stroke volume following cyclic changes in venous return [19]. They concluded that changes in intrathoracic pressure, rather than changes in tidal volume as such, determine dPP and SVV.

Likewise, the use of dynamic variables to estimate volume status during spontaneous breathing relies on swings in intrathoracic pressure sufficiently large to produce notable changes in stroke volume over one respiratory cycle if the heart is working on the steep part of the Frank-Starling curve. Several maneuvers can amplify intrathoracic pressure changes during spontaneous breathing, such as deep, forced inspiration, or in- or expiration against a resistance. During deep spontaneous inspiration, RAP decreases and the gradient for venous return increases, increasing right ventricular filling. In a preload responsive heart, this leads to an increase in stroke volume which is visible as maximal pulse pressure after the pulmonary transit time of 2–3 s, normally during expiration. During mechanical ventilation, intrathoracic and right atrial pressures increase in the inspiratory phase, which reduces the gradient for venous return and thus right ventricular filling. This leads to reduced left ventricular stroke volume and minimum pulse pressure a few heart beats later; normally during expiration. As these are opposite effects, a combination of the two forms of ventilation may neutralize the pressure changes specific to each respiratory mode, and reduce pulse pressure variations. We believe this explains why an increase in PEP-, but not CPAP-level, affected dPP in the present study. PEP increases intrathoracic pressure during spontaneous expiration, and the gradient for venous return decreases [20]. By contrast, CPAP increases airway pressures both during in- and expiration. This augments the reduction in preload and stroke volume during expiration, but the increase in venous return which normally occurs during spontaneous inspiration is offset by increasing intrathoracic pressure following insufflation of air under pressure. As a result, the maximal stroke volume and thus the variation in pulse pressure are smaller than when only an expiratory resistance is applied. This is illustrated by the observed changes in CVP (Fig 4). The difference between the peaks and troughs in CVP (dCVP) at any given LBNP-level tended to increase with increasing PEP, as only the maximal (expiratory) values increased. By contrast, during CPAP, the differences are smaller as both peaks and troughs tended to increase with increasing CPAP-levels, reflecting the continuously increased intrathoracic pressure.

Dynamic variables have shown some ability to diagnose hypovolemia or fluid responsiveness in spontaneously breathing subjects by manipulating respiratory pattern using Valsalva maneuvers [6], slow patterned breathing [21], a deep inspiratory maneuver [22] or forced inspiratory breathing [23]. However, in patients with hemodynamic instability, a forced respiratory maneuver decreased the diagnostic ability of dPP [24]. Airway pressures have also been amplified during spontaneous breathing by applying various respiratory resistors. In a study on healthy volunteers using the LBNP-model, a good diagnostic ability for stroke volume variation was found during spontaneous breathing, but stroke volume variation was actually reduced with hypovolemia [25]. The diagnostic ability, and also the reduction with hypovolemia, was abolished by applying supported ventilation. On the other hand, in a porcine model, dynamic variables predicted fluid responsiveness well using an expiratory resistor of 7.5 cmH_2_O [5]. In a setup with the similar respiratory resistors on healthy volunteers inducing central hypovolemia using head-up tilt [26], the best ability to diagnose hypovolemia was found for systolic pressure variations using both inspiratory and expiratory resistors of 7.5 cmH_2_O. However, inspiratory resistors are rarely, if ever, used in routine clinical practice, and when using only an expiratory resistor, a statistically significant diagnostic ability was found for SVV, but not for dPP [26]. Also using head-up tilt, a diagnostic ability of dPP to detect hypovolemia was found using expiratory resistors of 7.5 cmH_2_O when combined with a respiratory rate of 6 breaths/min [27]. These studies indicate that using only an expiratory resistor (PEP) of less than 10 cmH_2_O may not impose sufficient intrathoracic pressure change to diagnose hypovolemia or fluid responsiveness unless further respiratory interventions such as increased tidal volume or reduced respiratory rate (often implying increased tidal volumes) are also applied. However, our results suggest that by applying PEP as high as 10 cmH_2_O, the need for further respiratory adjustments may be reduced.

The photoplethysmographic waveform from which dPOP is calculated is complex, reflecting absorption of light from arterial, capillary and venous blood. Thus it is affected by both the cardiovascular, respiratory and autonomic nervous systems [28], and has been used to extract information about both fluid responsiveness [29], respiration [30] and pain [31]. dPOP appears to reflect volume status less consistently than dPP during mechanical ventilation, which is generally explained by the complexity of the signal as well as proprietary processing and filtering algorithms [29, 32]. This may also explain why the transition to PEP 10 was reflected in dPP, but not in dPOP in the present study, and why dPOP remained unchanged with increasing LBNP-level during baseline PEP, whereas dPP increased. In a previous study, our group found a significant association between LBNP-level and dPP, but not dPOP [33], but this study was limited by small sample size. dPOP and the related automated variable Pleth Variability Index (PVI) have been found to decrease in response to passive leg raise. However, correlations with changes in cardiac index and the ability to predict fluid responsiveness were weak [34–36]. PVI was also found to increase with LBNP -40 mmHg when both PEEP 5 cmH_2_O and tripled tidal volume was applied, but not with either in isolation [37]. The fact that dPOP reflected volume loss in the present study may indicate that, as for dPP, amplification of pleural pressure swings improves the ability of dPOP to reflect hypovolemia.

### Methodological considerations

As this study was performed on healthy volunteers, its findings may not be valid for all patients. Breathing through a resistor requires patient cooperation, and the effect on intrathoracic pressure at a given resistance may differ in and between patients according to respiratory effort. Differences in respiratory and heart rate may also affect the impact of a forced respiratory maneuver on dPP and dPOP, as fewer heart beats per respiratory cycle give fewer stroke volumes in which to detect a difference [38]. We did not standardize respiratory rate, as we wanted to investigate the effects of CPAP and PEP during conditions that approximate ordinary clinical use of PEP and CPAP. Baseline PEP and CPAP-levels of 0 cmH_2_O may not be regarded as regular spontaneous breathing, as breathing trough a mouthpiece or facemask as such may affect respiratory pattern. PEP and CPAP 0 cmH_2_O should rather be regarded as physiological baselines not intended for use in clinical practice, reflecting the experimental nature of this study.

The different respiratory interventions were of limited duration (5–6 respiratory cycles) as we feared that the advantages of longer intervention periods would be offset by earlier termination of the protocol due to exhaustion in some subjects. Increasing the number of breaths against a given resistance could have given more robust results. Individual LBNP-tolerance differs due to several factors [39]. Only 5 subjects completed LBNP -80 in the present study, reducing the amount of data at high LBNP-levels. However, we are not aware of mechanisms by which this would bias the estimates. At each LBNP-level, 1 minute was allowed for hemodynamic stabilization before respiratory interventions were started. However, we cannot exclude further stabilization also after this period. Therefore, the order of PEP- and CPAP-levels were randomized and alternated to account for a potential systematic effect of time.

For calculation of cardiac output, diameter of the aortic orifice and angle of insonation were not measured, but assumed. This probably led to a loss of accuracy, but as our analyses are based on changes and relative values of cardiac output, we do not believe this has had significant impact on our results.

To minimize the risk of complications in healthy volunteers, CVP was only measured in 10 subjects who had a prominent cubital vein entering the basilic vein, and insertion appeared technically uncomplicated. However, based on the estimates with confidence intervals, measurements from 10 subjects appear to suffice to illustrate the impact of different respiratory modes on CVP.

Calculations of the dynamic variables and dCVP were performed semi-automatically in R to increase reproducibility, as described in the S1 and S2 Files. As the algorithm has not been previously validated, all values were plotted and manually inspected to approximate the validity of manual calculation.

Different pulse oximeters use proprietary algorithms and produce different photoplethysmographic waveforms. Thus, dPOP from different pulse oximeters may give different results [40]. dPP was derived by the volume-clamp method, which is based on the photoplethysmographic technology. The photoplethysmographic technology is susceptible to disturbances caused by changes in vasomotor tone [41]. Good agreement has been found between pulse pressure variations obtained from non-invasive and intra-arterial blood pressure curves [42], however, dPP calculated from an invasively measured arterial pressure waveform may differ from that of the present study.

### Conclusion

In this study, we found that dPP and dPOP reflected progressive hypovolemia during all levels of CPAP and PEP, except for dPOP at baseline PEP. The application of PEP 10 cmH_2_O amplified the increase in dPP, but not in dPOP. Both progressive LBNP and the transition from baseline PEP to PEP 5 cmH_2_O and 10 cmH_2_O were reflected in dCVP. Clinical studies may elucidate whether respiratory changes in pulse pressure or the photoplethysmographic waveform amplitude reflect volume status in patients during treatment with PEP or CPAP.

## Supporting information

S1 File Calculation of dPP and dPOP. (DOCX)

S2 File Calculation of dCVP. (DOCX)

S3 File [Data_script.zip]. (ZIP)
