# Supplementary material for: Respiratory variations in pulse pressure and photoplethysmographic waveform amplitude during positive expiratory pressure and continuous positive airway pressure in a model of progressive hypovolemia
Source: PLoS One. 2019 Sep 27;14(9):e0223071. doi: 10.1371/journal.pone.0223071 (PMC6764667; doi:10.1371/journal.pone.0223071)
Supplement: S1 File — (DOCX) [file pone.0223071.s001.docx]

**S1 File. Calculation of dPP and dPOP_._**

Arterial pressure and photoplethysmographic waveforms were exported as .txt-files and imported to R using RStudio.

The signals from the two sampling softwares (Regist3 and SignalExpress) were time-synchronized and given a common time-stamp.

The R-peaks of the ECG were detected and manually inspected with code using the “wavelets”-package.

To locate the peaks within each heartbeat, a smoothed curve was created using the “analyze.wavelet” and “reconstruct”-functions of the “WaveletComp”-package with a bandpass filter restricted to between 0.7 and 1.3 times the heart rate. The peak of this smoothed curve was located using the “peakpick”-function of the “peakPick”-package.

The peak of the original signal was found as the maximal value of the original waveform within ±0.3 s of the peak of the smoothed signal. The trough of the original signal was found as the minimal value within 0.4 s before the peak. Pulse pressure and photoplethysmographic amplitude was calculated as the difference between this maximal and minimal value.

Respiration was registered from the thorax impedance of the ECG-leads from the GE-Solar monitor. After smoothing the respiration signal using the “analyze.wavelet”-function with lower and upper periods of 2 and 20 s, respectively, the smoothed curve was plotted with the original, and lower and/or upper periods were adjusted if deemed necessary after visual inspection. The peaks of the smoothed signal were detected with the “peakpick”-function. Maximal and minimal amplitudes for calculation of dPP and dPOP were identified between two peaks of the smoothed respiration signal.

All results were plotted and manually inspected before being entered to the final dataset with obviously erroneous values being removed or manually corrected.

**
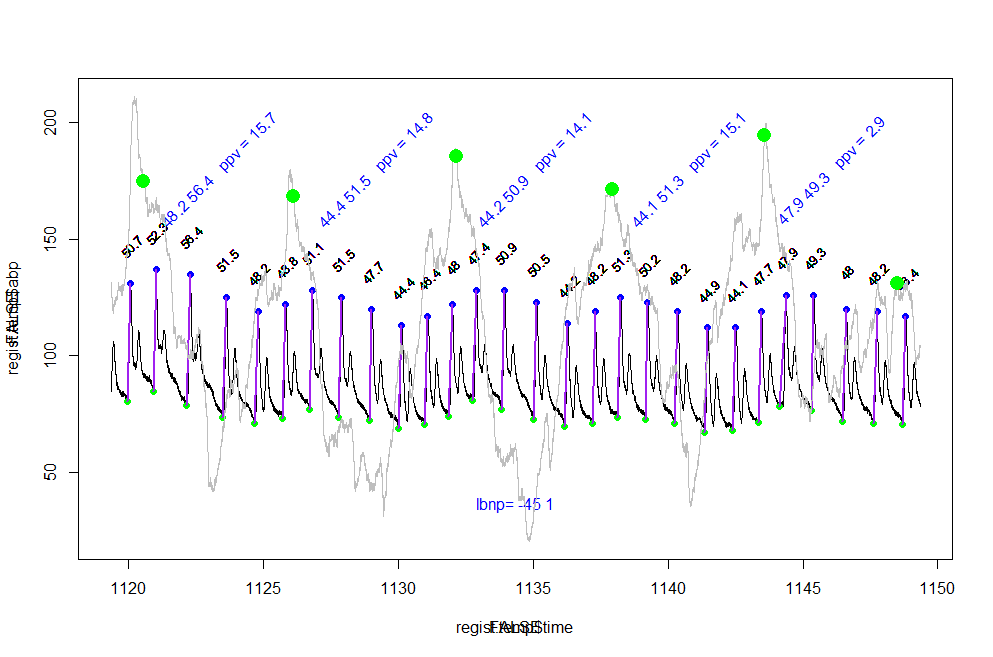
**

**Supplementary Figure 1:** Example of the algorithm for calculating respiratory variations in pulse pressure from the Finometer, in this case for PEP 5 cmH_2_O at LBNP 40 mmHg. The black line is the original arterial pressure waveform. The peaks were found by locating the peaks of a smoothed signal (not shown), and thereafter locating and quantifying the peaks of the original waveform, presented as blue dots. The troughs of the original signal were found thereafter, presented as small green dots. The pulse pressure of each heartbeat was calculated as the difference between these two, presented as black numbers. The respiratory cycles were delimitated by finding the peaks of the respiratory signal of the ECG-leads, presented as large green dots. The smallest and largest pulse pressures between two respiratory peaks were found, and pulse pressure variations calculated, presented as blue numbers. The values not representing respiratory cycles with resistance were removed after manual inspection. Time in seconds on the x-axis, arterial blood pressure in mmHg on the y-axis (labels from several plots overprojected in the example). dPOP was calculated using a corresponding algorithm.
