# Supplementary material for: Respiratory variations in pulse pressure and photoplethysmographic waveform amplitude during positive expiratory pressure and continuous positive airway pressure in a model of progressive hypovolemia
Source: PLoS One. 2019 Sep 27;14(9):e0223071. doi: 10.1371/journal.pone.0223071 (PMC6764667; doi:10.1371/journal.pone.0223071)
Supplement: S2 File — (DOCX) [file pone.0223071.s002.docx]

**S2 File. Calculation of dCVP.**

Central venous pressure waveforms were exported as .txt-files and imported to R using RStudio. The signal was downsampled from 400 to 40 Hz.

To calculate the variations with respiration, the signal was smoothed using the “analyze.wavelet” and “reconstruct”-functions of the “WaveletComp”-package with a lower period set to 2 s. Peaks and troughs were located using the “peakpick” of the “peakPick” package.

All results were plotted and manually inspected before being entered to the final dataset with obviously erroneous values being removed or manually corrected. An example is presented below.


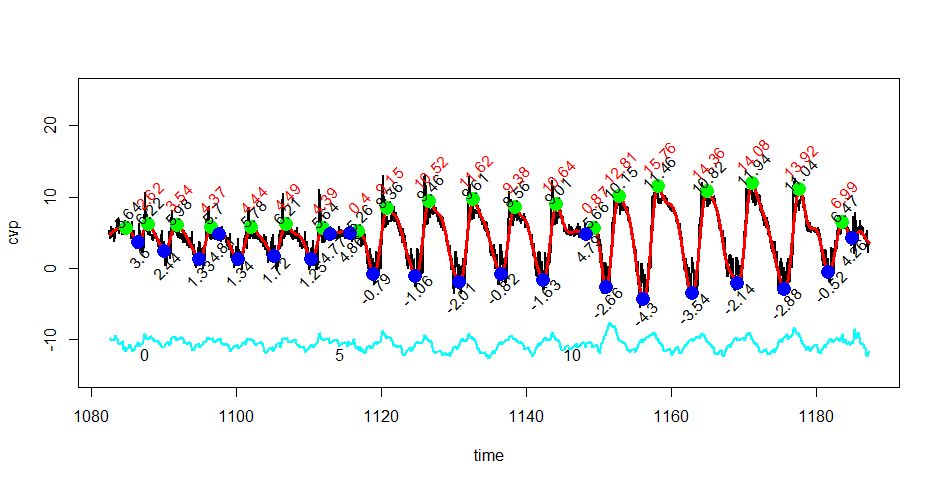


**Supplementary Figure 2:** Example of the algorithm for calculating respiratory variations in CVP. The black line is the CVP-waveform downsampled to 40 Hz. The red line is the smoothed CVP-waveform after wavelet analysis and reconstruction. The local minima and maxima for each respiratory cycle are blue and green dots with values as presented, respectively. The difference between these values, representing the respiratory variation in CVP, is presented in red. The cyan line is respiration from the ECG-leads. The numbers below the cyan line are comments entered during the experiments representing the respiratory resistance in cmH_2_0, in this case, the level of expiratory resistance. The values not representing respiratory cycles with resistance were removed after manual inspection. Time in seconds on the x-axis, CVP in mmHg on the y-axis.
